# Comparison of Copper Scavenging Capacity between Two Different Red Mud Types

**DOI:** 10.3390/ma5091708

**Published:** 2012-09-24

**Authors:** Yingqun Ma, Chunhua Si, Chuxia Lin

**Affiliations:** 1 Centre for Ecological and Environmental Technologies, South China Agricultural University, Guangzhou 510642, China; E-Mails: myoungking@163.com (Y.M.); siqisxnd@yahoo.com.cn (C.S.); 2 Australian Centre for Sustainable Catchments, University of Southern Queensland, Toowoomba QLD 4350, Australia; 3 Chinese Research Academy of Environmental Sciences, Beijing 100012, China

**Keywords:** alumina, bauxite, red mud, calcite, alkaline, copper, boehmite, atacamite, metal immobilization

## Abstract

A batch experiment was conducted to compare the Cu scavenging capacity between two different red mud types: the first one was a highly basic red mud derived from a combined sintering and Bayer process, and the second one was a seawater-neutralized red mud derived from the Bayer process. The first red mud contained substantial amounts of CaCO_3_, which, in combination with the high OH^−^ activity, favored the immobilization of water-borne Cu through massive formation of atacamite. In comparison, the seawater-neutralized red mud had a lower pH and was dominated by boehmite, which was likely to play a significant role in Cu adsorption. Overall, it appears that Cu was more tightly retained by the CaCO_3_-dominated red mud than the boehmite-dominated red mud. It is concluded that the heterogeneity of red mud has marked influences on its capacity to immobilize water-borne Cu and maintain the long-term stability of the immobilized Cu species. The research findings obtained from this study have implications for the development of Cu immobilization technology by using appropriate waste materials generated from the aluminium industry.

## 1. Introduction

As an abundant waste material generated from alumina refining, beneficial utilization of red mud is a viable option to reduce the amount of this hazardous material that requires costly containment facilities for its safe disposal [1,2,3,4,5,6,7,8,9,10,11]. The uses of red mud or modified red mud for treating acidic, heavy metal-bearing soils and wastewater are among the proposed applications [12,13,14,15,16,17,18,19,20,21,22,23,24].

In a previous study [22] to investigate the competitive removal of water-borne Cu, Zn and Cd by a red mud sample collected from the Zhengzhou Alumina Refinery, we found that the water-borne Cu had a higher affinity to the red mud in the presence of chloride, as compared to the water-borne Zn and Cd. The major mechanism responsible for the preferential retention of Cu by the red mud was the formation of atacamite (Cu_2_(OH)_3_Cl), which maximized the Cu scavenging effect.

Red mud is a heterogeneous material and its characteristics varies markedly from place to place, depending on the sources of bauxite ores, alumina refining processes and the methods used for red mud disposal [25,26]. Consequently, the capacity of red mud to scavenge Cu may also vary from red mud type to red mud type. In the current study, two significantly different red mud types were used to test their capacities to remove water-borne Cu. Fractionation of the retained Cu was also investigated. The objective was to understand the influences of red mud heterogeneity on its performance as a Cu scavenger under the set experimental conditions.

## 2. Materials and Methods

### 2.1. The Red Mud Samples

Two different red mud types were used for the experiment. The first one (labeled as GR) was collected from the Guizhou Alumina Refinery at Guiyang, China and the second one (Labeled as QR) was collected from the Queensland Alumina Refinery at Gladstone, Australia. The Guizhou Alumina Refinery used diaspore-dominated bauxite ore as the feedstock [27]. A combined sintering process and Bayer Process method was used for bauxite processing and the red mud was disposed of using a wet disposal method. The Queensland Alumina Refinery used a Bayer Process method for alumina extraction from gibbsite-boehmite type bauxite ore mined from Weipa, northern Queensland [28]. The red mud was treated by seawater before being thickened and deposited as slurry in the disposal facility.

Surface red mud samples were collected from the red mud storage facilities of the above two refineries. In the laboratory, the red mud samples were air-dried and ground to pass through a 60 mesh sieve (250 µm). Some chemical characteristics of the two red mud samples are given in Table 1.

### 2.2. Copper Scavenging Experiment

The experiment was performed in triplicate. For each red mud type, 25 grams of the red mud sample were reacted with a series of CuCl_2_ solutions with varying concentrations. A total of eight Cu concentration levels were originally set for the experiment: 3437.5, 6875, 13,750, 27,500, 34,375, 41,250, 48,125 and 61,875 mg/L. Pre-experiment test showed that the QR was not able to remove all water-borne Cu at a dosage level of 27,500 mg/L. Therefore, only five Cu concentration levels were performed for the QR: 3437.5, 6875, 13,750, 27,500, 34,375 mg/L. The red mud sample was mixed with 100 mL of a relevant solution in a stoppered conical flask (150 mL) and shaken on a HY-4 horizontal shaker for 16 h. The suspension was then transferred to a centrifuge tube for centrifugation at 4000 rpm for 10 min. After separation, the supernatant was used for determination of soluble Cu, Ca and Fe. The solid residue was used for various chemical and mineralogical analyses after washing with deionized water twice and air-drying.

**Table 1:** Some major chemical and mineralogical parameters of the two red mud types used in the experiment.

Parameter	Guizhou red mud	Queensland red mud
**pH**	11.0	9.4
**EC (dS/m)**	0.89	1.26
**Total carbon (%)**	4.13	1.05
**Total Ca (mg/kg)**	183,800	61,900
**Total Cu (mg/kg)**	85	145
**Soluble K (mmol/kg)**	6.24	16.9
**Soluble Na (mmol/kg)**	70.2	71.9
**Soluble Ca (mmol/kg)**	1.19	0.36
**Soluble Mg (mmol/kg)**	0.06	0.11
**Exchangeable K (mmol/kg)**	83.7	74.0
**Exchangeable Na (mmol/kg)**	218	236
**Exchangeable Ca (mmol/kg)**	341	23.7
**Exchangeable Mg (mmol/kg)**	4.01	4.16
**Major minerals**	calcite, perovskite, monohydrocalcite, magnetite	boehmite, quartz, larnite, calcite, magnetite, perovskite, gibbsite, sodalite, anatase

### 2.3. Analytical Methods

Various fractions of Cu in the solid samples were extracted by different extractants. 1:5 (red mud:deionized water) and 1:5 (red mud:1 M NH_4_Cl) extracts were prepared for determinations of the water-extractable and the 1 M NH_4_Cl-extractable Cu. The water-extractable fraction was used to estimate the concentration of the water-soluble Cu fraction. The 1 M NH_4_Cl-extractable fraction includes water-soluble and exchangeable Cu. The improved BCR sequential extraction procedure [29] was used to separate the following three Cu fractions in the solid residues: (a) 0.11 M HCH_3_COO-extractable Cu (termed as Fraction I), (b) 0.5 M NH_2_OH·HCl-extractable Cu (termed as Fraction II) and (c) 1 M NH_2_CH_3_COOH-extractable Cu after 30% H_2_O_2_ digestion (termed as Fraction III). In the BCR system, Fraction I was thought to include water-soluble, adsorbed and carbonate-bound metal; Fraction II was viewed to be in the forms bound to oxides of iron and manganese; and Fraction III was believed to include a metal bound to organic matter and sulfide minerals. The BCR sequential extraction procedure was designed for fractionation of heavy metals in soils and sediments. It was adopted for this study because no verified sequential extraction methods for red mud materials were available. However, it was realized that the operationally defined fractions of heavy metals obtained from the BCR sequential extraction procedure may require new interpretation when the method was used for heavy metal fractionation of red mud, which is somewhat different from soils or sediments in terms of physico-chemical properties and composition. Total Cu were extracted by digestion of a sample with a HF/HNO_3_/HClO_4_ mixed solution.

The concentrations of Cu, Ca and Fe in the supernatants and various extracts of the solid residues were determined by atomic absorption spectrometry (AAS). Carbon concentration of the solid residues was measured by a LECO CNS Analyzer. Mineral composition was determined using a Bruker D8 ADVANCE X-ray diffractometer. The Materials Data Inc. software Jade 5.0 was used for phase identification. Semi-quantitative phase analysis was performed using the computer program PCPDFWIN (I/Icor reference intensity ratio method). The samples were also used for examination of micro-morphological characteristics by A FEI-XL30 environmental scanning electron microscope coupled with energy dispersive X-ray spectrometer (ESEM/EDS).

### 2.4. Statistical Method

The data for the replicated experiment are presented as mean ± SD. The significant treatment differences were tested using a Duncan's multiple range test method.

## 3. Results

### 3.1. Concentrations of Cu, Fe and Ca in the Solutions after 16 h Reaction

After shaking of the red mud-CuCl_2_ solution mixtures for 16 h, almost all the added Cu was removed from the solution for the Guizhou red mud (GR). The highest dose of Cu in this experiment was 61,875 mg/L, indicating that GR had a Cu scavenging capacity greater than 247 g/kg. In comparison, greater than 7% and 19% of the added Cu remained in the solution for the Queensland red mud (QR) when the initial concentration of solution Cu was 27,500 and 34,375 mg/L, respectively (Table 2).

**Table 2:** Concentrations of Cu, Fe and Ca in the reacting solutions after 16 h reaction for the two red mud types.

Red mud type	OCCRS (mg/L)	Cu (mg/L)	Fe (mg/L)	Ca (mg/L)
**GR**	3437.5	0.05 ± 0.01a	0.04 ± 0.00ab	935.6 ± 21.17a
	6875	0.04 ± 0.00a	0.03 ± 0.01a	2294 ± 268.1b
	13,750	0.07 ± 0.07a	0.04 ± 0.01ab	5623 ± 133.2c
	27,500	0.05 ± 0.01a	0.04 ± 0.01ab	11,482 ± 256.70d
	34,375	0.30 ± 0.37a	0.49 ± 0.01ab	14,128 ± 41.36e
	41,250	0.11 ± 0.02a	0.05 ± 0.00ab	17,274 ± 267.1f
	48,125	0.13 ± 0.02a	0.05 ± 0.01ab	19,520 ± 476.9g
	61,875	0.12 ± 0.01a	0.08 ± 0.06b	27,407 ± 598.2h
**QR**	3437.5	1.46 ± 0.19a	0.04 ± 0.00a	536.2 ± 15.31a
	6875	1.40 ± 0.68a	0.05 ± 0.02a	1281 ± 194.5b
	13,750	2.23 ± 0.29a	0.05 ± 0.00a	3484 ± 22.45c
	27,500	1988 ± 168.1b	0.12 ± 0.07b	5593 ± 361.7d
	34,375	6748 ± 182.0c	0.10 ± 0.02b	5781 ± 190.1d

Notes: OCCRS: Original Cu concentration in the reacting solution; Means with different letters in the same column differ significantly at P < 0.05.

With the increase in Cu dose, the concentration of Ca in the solution increased for both red mud types. This was consistent with what was observed in our previous study [22]. However, Ca concentration was always higher in GR than in QR at the same Cu dose level (Table 2).

At any dose of CuCl_2_, only trace amounts of Fe was detected in the solutions of either GR or QR after 16 h reaction (Table 2).

### 3.2. Total Retained Cu and Total Carbon in the Solid Residues

**Figure 1:**
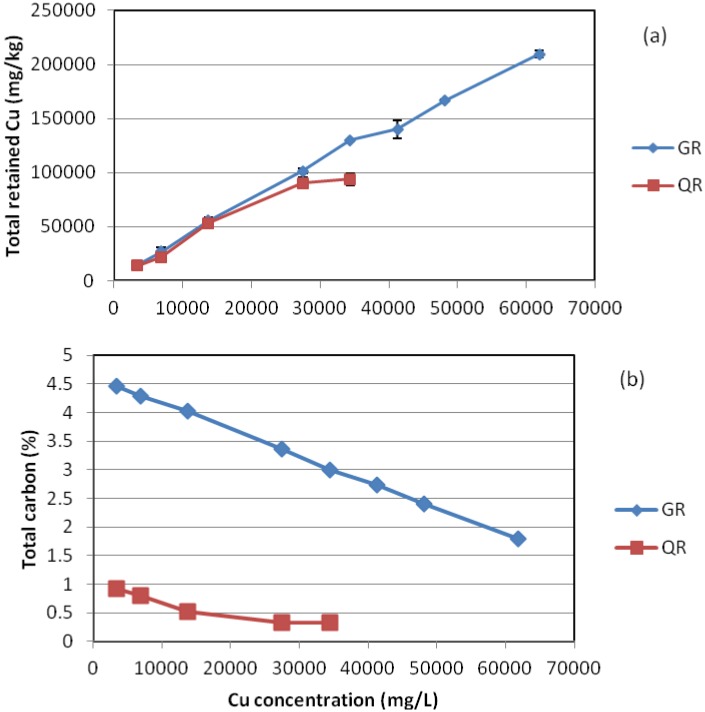
Diagrams showing the change in (**a**) the total retained Cu and (**b**) the total carbon in the solid residues with increasing dose of CuCl_2_ for the two tested red mud types.

Change in the total retained Cu with the increase in CuCl_2_ dose for the two red mud types is shown in Figure 1a. For GR, the total retained Cu increased nearly linearly with increasing dose for the examined range of CuCl_2_ concentrations. Initially, the dose-response relationship for QR was highly consistent with that for GR. However, a gap was created at least before a Cu dose of 27,500 mg/L was reached; QR had a lower total retained Cu value than did GR. There was no marked change in the total retained Cu between the Cu dose of 27,500 mg/L and 34,375 mg/L.

The change in the total carbon content in the solid residues with increasing Cu dose showed an opposite trend to that of the total retained Cu. Similarly, a nearly linear dose-response relationship was observed for GR while QR exhibited a relatively rapid, gentle and insignificant change in the dose ranges of 3437.5–13,750, 13,750–27,500 and 27,500–34,375 mg/L, respectively (Figure 1b).

### 3.3. pH, EC, Water-Extractable and NH_4_Cl-Extractable Cu of the Solid Residues

There was a trend that pH decreased and EC increased with increasing dose of CuCl_2_ for both red mud types. For GR, the mean pH decreased from 9.12 to 7.60 with the increase in Cu dose from 3437.5 to 61,875 mg/L. For QR, the pH was below 5 at a Cu dose greater than 27,500 mg/L (Table 3).

**Table 3:** pH, EC, water-extractable and NH_4_Cl-extractable Cu in the solid residues after 16 h reaction.

Red mud type	OCCRS (mg/L)	pH	EC (dS/m)	Cu_w_ (mg/kg)	Cu_am_ (mg/kg)
**GR**	3437.5	9.12 ± 0.02e	0.198 ± 0.003bc	0.23 ± 0.10ab	275 ± 28.2d
	6875	8.79 ± 0.04d	0.189 ± 0.008b	0.14 ± 0.05ab	286 ± 7.97d
	13,750	8.46 ± 0.07c	0.174 ± 0.003a	0.50 ± 0.08c	145 ± 13.4c
	27,500	8.00 ± 0.05b	0.204 ± 0.009c	0.27 ± 0.15ab	91.3 ± 4.55b
	34,375	7.91 ± 0.06b	0.222 ± 0.006d	0.09 ± 0.07a	81.7 ± 8.53b
	41,250	8.00 ± 0.20b	0.222 ± 0.005d	0.09 ± 0.02a	85.9 ± 7.37b
	48,125	7.73 ± 0.16a	0.233 ± 0.008e	0.27 ± 0.19ab	71.6 ± 1.26ab
	61,875	7.60 ± 0.03a	0.286 ± 0.001f	0.33 ± 0.16bc	55.0 ± 4.32a
**QR**	3437.5	7.99 ± 0.03d	0.214 ± 0.003a	0.02 ± 0.02a	198 ± 12.66d
	6875	7.95 ± 0.01d	0.215 ± 0.003a	0.04 ± 0.02a	181 ± 1.87c
	13,750	7.66 ± 0.06c	0.228 ± 0.005b	0.04 ± 0.02a	92.5 ± 9.10a
	27,500	4.82 ± 0.19b	0.253 ± 0.012c	51.9 ± 3.41b	108 ± 3.06b
	34,375	4.64 ± 0.04a	0.358 ± 0.001d	186 ± 5.22c	196 ± 5.13ab

Notes: OCCRS: original Cu concentration in the reacting solution; Cu_w_: water-extractable Cu; Cu_am_: NH_4_Cl-extractable Cu; Means with different letters in the same column differ significantly at P < 0.05.

For GR, the water-extractable Cu (Cu_w_) was all very low regardless of the dosage level of CuCl_2_. In contrast, Cu_w_ in QR was >50 mg/kg at a Cu dose greater than 27,500 mg/L. Extraction by ammonium chloride enhanced the release of the retained Cu. This was particularly true for the treatments with lower doses of CuCl_2_ (Table 3).

### 3.4. Ca- and Cu-Bearing Minerals in the Solid Residues

**Figure 2:**
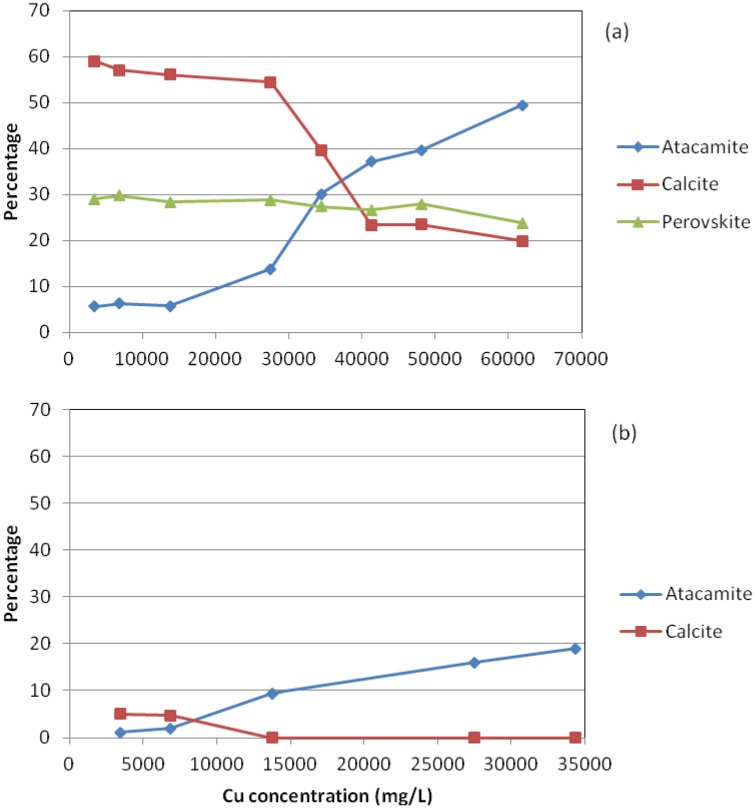
Changes in the abundance of Ca- and Cu-bearing minerals with increasing Cu dose for (**a**) GR and (**b**) QR.

For GR, the abundance of atacamite showed no marked increase in the Cu dose range of 3437.5–13,750 mg/L; an increase in atacamite occurred when the Cu dose was increased to 27,500 mg/L; the abundance of atacamite sharply increased from the Cu dose of 27,500 mg/L to the Cu dose of 41,250 mg/L, followed by a relatively gentler increase in the Cu dose range of 41,250–61,875 mg/L. This dose-response trend was accompanied by an opposite dose-response trend of calcite. In contrast with calcite, perovskite displayed no marked change despite that a general trend showing slight decrease in the abundance of perovskite with increasing Cu dose was observed (Figure 2a).

No perovskite was detected for QR. Similar to GR, change in either atacamite or calcite was not remarkable in the low Cu dose range. The abundance of atacamite markedly increased, accompanied by a marked decrease in calcite when the Cu dose was increased from 6875 mg/L to 13,750 mg/L. After this, atacamite increased slowly with increasing Cu dose. Calcite was not detected in the high Cu dose range (13,750–34,375 mg/L).

### 3.5. Cu Fractionation

**Figure 3:**
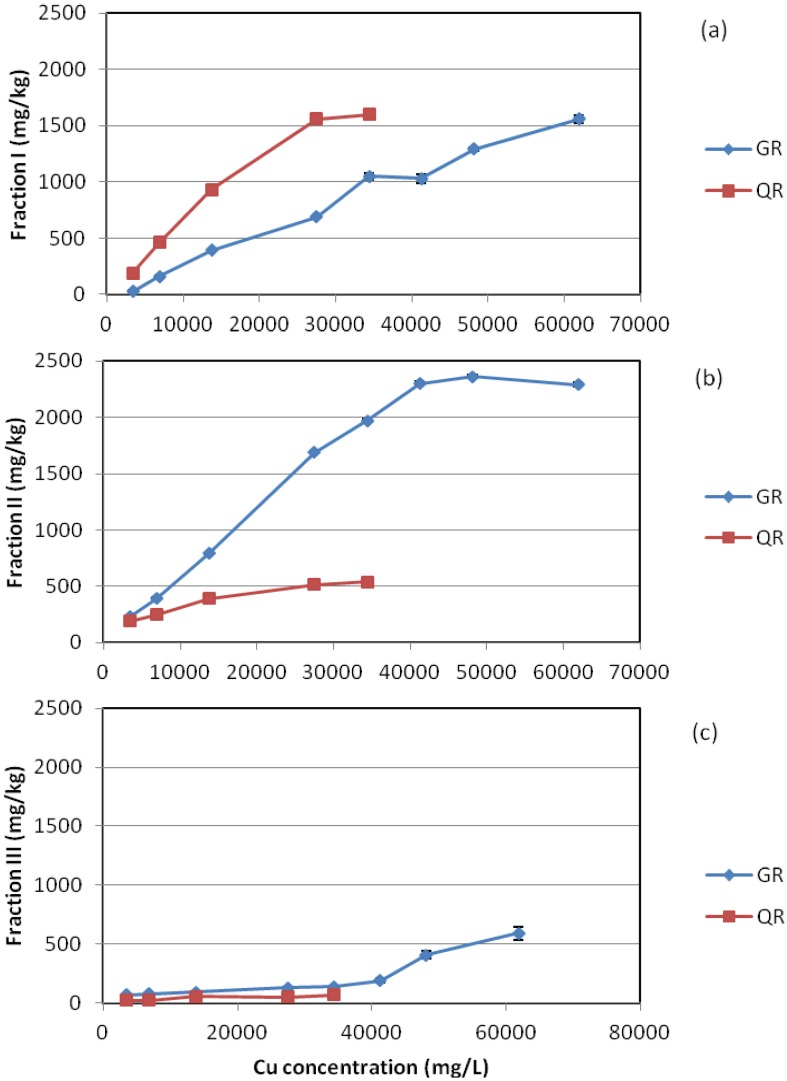
Comparison of the variation trend of various Cu fractions between GR and QR: (**a**) Fraction I; (**b**) Fraction II; (**c**) Fraction III.

The distribution of three operationally defined Cu fractions was different between the two red mud types. The order of various Cu fractions was: Fraction II > Fraction I > Fraction III for GR and Fraction I > Fraction II > Fraction III for QR (Figure 3). For individual fractions, Fraction I was higher in QR than in GR; Fraction II was much higher in GR than in QR; and Fraction III was slightly higher in GR than in QR.

### 3.6. SEM Observation and EDS Analysis

**Figure 4:**
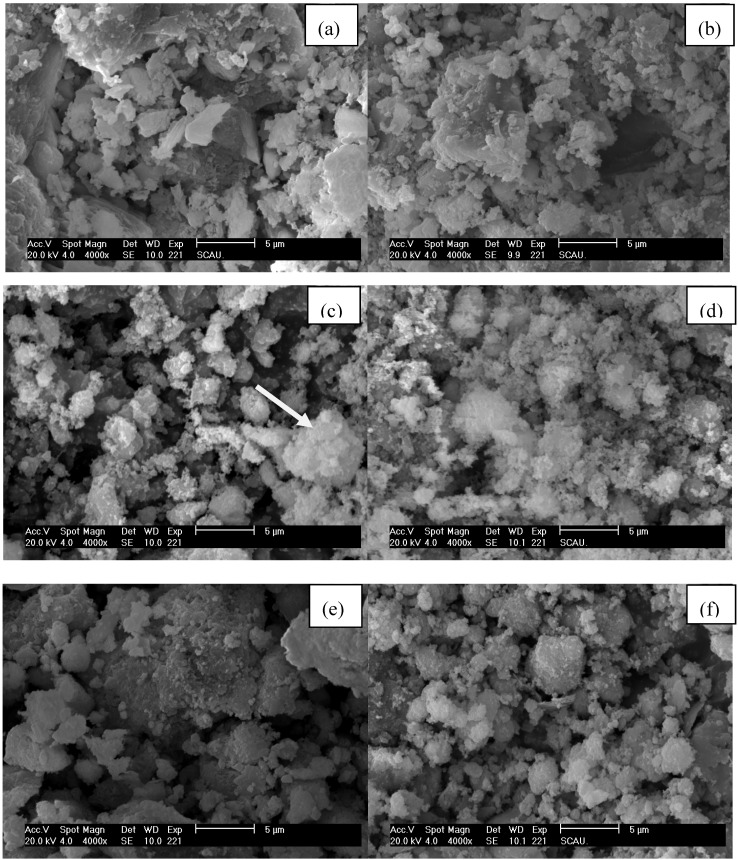
SEM images showing micro-morphological characteristics of (**a**) original GR; (**b**) GR at a Cu dose of 27,500 mg/L; (**c**) GR at a Cu dose of 61,875 mg/L; (**d**) blue precipitates for GR at a Cu dose of 61,875 mg/L; (**e**) original QR; (**f**) QR at a Cu dose of 34,375 mg/L.

The original GR and QR consisted predominantly of densely packed aggregates (Figure 4a and e). Reaction with CuCl_2_ resulted in the formation of loose aggregates (Figure 4b and f), and the abundance and size of such loose aggregates tended to increase with increasing Cu dose (Figure 4c).

**Figure 5:**
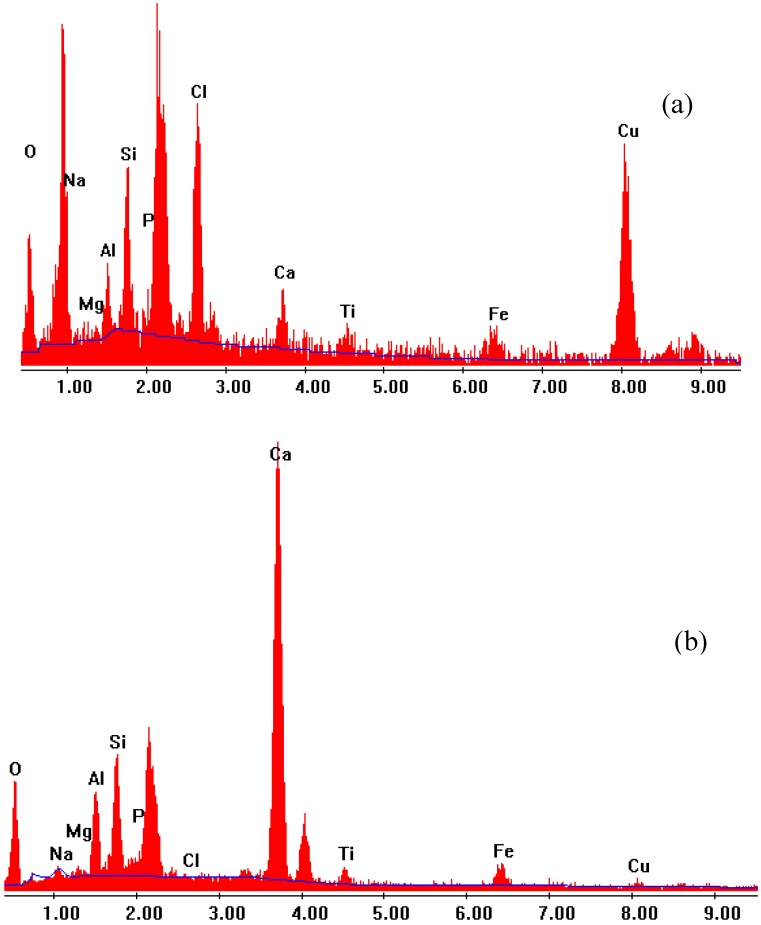
EDS graphs showing peaks of major elements detected for (**a**) blue precipitates (at Cu dose of 61,875 mg/L) and (**b**) the original GR sample.

There were some blue precipitates on the wall of the conical flask for GR at high Cu doses. These precipitates appeared as loose, rough and irregular aggregates of varying sizes (Figure 4d). EDS analysis showed that these materials had markedly elevated concentration of Cu and Cl and reduced concentration of Ca (Figure 5a), as compared to the original GR (Figure 5b).

## 4. Discussion

The two red mud types investigated in this study were remarkably different in terms of chemical and mineralogical characteristics. GR was a CaCO_3_-dominated, highly basic red mud while QR was a boehmite-dominated and less basic red mud. Due to the presence of substantial amounts of NaOH in GR, the initial formation of atacamite was likely to be through the following reaction [22]:

2CuCl_2_ + 3NaOH → Cu_2_(OH)_3_Cl + 3NaCl (1)

The above reaction did not involve dissolution of calcite. This explains why the carbon content in the soil residue did not decrease in the lower Cu dose range (3437.5–6875 mg/L). The even higher carbon content in the solid residues, as compared to that in the original red mud, can be attributed to the mass loss of the solid material as a result of dissolution of the soluble constitutes when they were in contact with the CuCl_2_ solution. This is further confirmed by the fact that the pH of these two solid residues had a pH > 8.7, indicating that there was still free OH^−^ in the solid residues. Dissolution of calcite at a pH > 8.3 was kinetically slow [30]. Therefore, reaction in Equation (2) was unlikely to take place to any significant degree. With the increase in the Cu dose, OH^−^ was eventually depleted and CaCO_3_ replace OH^−^ to react with CuCl_2_, as expressed below:

2CuCl_2_ + 2CaCO_3_ + 2H_2_O → Cu_2_(OH)_3_Cl + 3Cl^−^ + OH^−^ + 2Ca^2+^ + CO_2_ (2)

This was well reflected in the scenarios of high Cu doses, showing lower carbon content in the solid residue, relative to that in the original red mud and a clear trend that carbon content in the solid residue decreased with increasing Cu dose (Figure 1b).

In contrast, the carbon content in the solid residue was lower than that in the original red mud for QR even at the lowest Cu dose (3437.5 mg/L). Since the amount of free OH^−^ in QR was very limited, reaction in Equation (2) took place immediately following the mixing of red mud with CuCl_2_. It is clear that the Cu-scavenging capacity of QR was almost depleted at a Cu dose of 27,500 mg/L. This can be attributed to the small amount of CaCO_3_ present in QR.

The difference in the distribution of the three Cu fractions suggests that the mineral composition of red mud had a marked influence on the binding form of Cu. The boehmite-dominated QR tended to have stronger capacity to hold Cu in the form of Fraction I, which consists of soluble, adsorbed and carbonate-bound Cu species. The pH of the QR residues was <5 when the Cu dose was >27,500 mg/L (Table 3). This explains the high soluble Cu concentration in the QR residues at high Cu doses because the solubility of Cu compounds tend to increase with decreasing pH. Since aluminium oxides are good adsorbents for Cu [31,32], the presence of substantial amounts of aluminium oxides/hydroxides (boehmite and gibbsite) might be responsible for the increased amounts of adsorbed Cu and consequently contributed significantly to the high proportion of Fraction I-Cu species in QR. The Fraction II-dominated regime for Cu in GR was attributable to the presence of considerable amounts of atacamite [22]. Fraction I-Cu species was believed to be more labile than Fraction II-Cu species [29]. Therefore, it appears that the water-borne Cu was more tightly bound by GR than QR. This suggests that the former is a better material than the latter in terms of its capacity to immobilize water-borne Cu and maintain long-term stability of the immobilized Cu species.

The Cu scavenging capacity of GR was over 247 g/kg, which was much greater than that reported for an alginate encapsulated magnetic sorbent (63 g/kg) [33] and other organic sorbents (3.9–16.4 g/kg) [34,35]. The extremely high capacity of GR to immobilize water-borne Cu was attributable to the precipitation of atacamite as a result of acid neutralization by the alkaline materials present in the red mud. This differs from the adsorption mechanisms dominated in the latter scenarios. It is possible that adsorption mechanisms were also involved in our experiment. However, the adsorption fingerprints in the Cu dose-response chart could be masked by the strong precipitation footprints.

Copper is commonly present in wastewater generated by mining, printed circuit board manufacturing, electronics plating, plating, wire drawing, copper polishing, paint manufacturing, wood preservatives and printing operations. The research findings obtained from this study have implications for developing innovative technologies to treat various Cu-containing wastewaters.

## 5. Conclusions

The highly basic, CaCO_3_-rich red mud had much stronger capacity than the seawater-neutralized, boehmite-dominated red mud in terms of scavenging Cu from solutions. It is also likely that the Cu was more tightly retained by the former than the latter. Based on these observations, it is concluded that the heterogeneity of red mud has marked influences on its capacity to immobilize water-borne Cu and maintain the long-term stability of the immobilized Cu species.
